# Greater vegetable variety and amount are associated with lower prevalence of coronary heart disease: National Health and Nutrition Examination Survey, 1999–2014

**DOI:** 10.1186/s12937-018-0376-4

**Published:** 2018-07-10

**Authors:** Zach Conrad, Susan Raatz, Lisa Jahns

**Affiliations:** 1 US Department of Agriculture, Agricultural Research Service, Grand Forks Human Nutrition Research Center, 2420 2nd Ave. N, Grand Forks, ND 58203 USA; 2 Department of Food Science and Nutrition, University of Minnesota, Saint Paul, MN 55108 USA

## Abstract

**Background:**

The 2015–2020 Dietary Guidelines for Americans (DGA) provides specific intake recommendations for vegetable variety and amount in order to protect against chronic disease. However, to the best of our knowledge, no studies have examined the link between DGA recommended vegetable variety and cardiometabolic disease. To address this research gap, our aim was to estimate the relationship between vegetable variety, vegetable amount, and prevalent cardiometabolic disease subtypes, and to assess potential determinants of vegetable variety.

**Methods:**

Data on food intake and reported cardiometabolic disease status were acquired for 38,981 adults from the National Health and Nutrition Examination Survey (1999–2014). Vegetable variety was measured using a modified dietary diversity index that was adjusted for the potential confounding effects of vegetable amount. Temporal trends in vegetable variety and amount were assessed using univariate linear regression models. Multivariate logistic regression models were used to estimate the relationship between vegetable variety and prevalent disease, and between vegetable amount and prevalent disease. Multivariate ordered logistic regression models were used to assess the relationship between vegetable variety and explanatory variables.

**Results:**

Overall, vegetable variety decreased (*P* = 0.035) from 1999 to 2014, but vegetable amount did not (*P* = 0.864). Intake of starchy vegetables decreased (*P* < 0.001), and intake of dark green vegetables increased (*P* < 0.001) over this 16-year period, but no trends were observed for other subgroups. An inverse linear relationship was observed between vegetable variety and prevalent coronary heart disease (*P*-trend = 0.032) but not other prevalent diseases; and between vegetable amount and coronary heart disease (*P*-trend = 0.026) but not other prevalent diseases. Individuals who reported consuming dark green vegetables had lower odds of having cardiovascular disease (0.86, 95% CI: 0.74–0.99) and coronary heart disease (0.78, 0.65–0.94) compared to individuals who reported not consuming any green vegetables. Living with a domestic partner was associated with greater vegetable variety (*P* = < 0.001), and currently smoking was associated with lower vegetable variety (*P* = < 0.001). Vegetable variety and amount were positively associated (*P* < 0.001).

**Conclusions:**

Vegetable variety and amount were inversely associated with prevalent coronary heart disease. Vegetable variety was strongly associated with vegetable amount, likely mediated by reduced habituation and increased liking. Increasing vegetable variety and amount are still important messages for the public.

**Electronic supplementary material:**

The online version of this article (10.1186/s12937-018-0376-4) contains supplementary material, which is available to authorized users.

## Background

Vegetables are good sources of micronutrients and phytochemicals that are associated with reduced risk for chronic disease [1]. Greater intake of vegetables has been consistently associated with reduced risk of mortality from cardiovascular diseases [2] like coronary heart disease [3] and stroke [4]. Accordingly, the Dietary Guidelines for Americans (DGA) 2015–2020 provides specific recommendations for daily vegetable intake depending on energy needs, with specific recommendations for subgroups, including dark green, red and orange, legumes, starchy vegetables, and other vegetables [5].

The health-promoting bioactive compounds in vegetables are not evenly distributed across different types, which is the reason that the DGA 2015–2020 also provides specific recommendations for variety of daily vegetable intake, meaning that vegetable intake should be consumed in prescribed proportions of different vegetable subgroups [5]. The development of these vegetable variety recommendations was informed by modelling approaches that demonstrated the optimal proportions of vegetable subgroups needed to meet Dietary Recommended Intakes (DRIs) for micronutrients, and to be achievable for consumers [6]. Dietary variety also prevents habituation and decreased liking, which can occur if the same or similar foods are consumed repeatedly [7, 8]. Increasing variety of vegetables in a single meal can increase liking of vegetables [9] and is positively associated with the amount of vegetables consumed [10–12].

Despite substantial evidence linking vegetable intake amount and risk of cardiometabolic health outcomes [2–4], few studies have examined the link between vegetable variety and cardiometabolic disease. Bhupathiraju et al. observed that fruit and vegetable intake amount, but not variety, was associated with reduced risk of coronary heart disease [13]. However, variety was measured as the number of different fruits and vegetables consumed per week rather than meeting the recommended vegetable subgroup proportions of the DGA. To the best of our knowledge, there are no studies that have assessed the relationship between adherence to the DGA 2015–2020 recommended vegetable intake variety and prevalence of cardiometabolic disease among the US adult general population. This is problematic because clinicians use the DGA to provide dietary recommendations on vegetable intake to their patients [14], but it is not known whether these recommendations are associated with positive health outcomes. This is an important research gap that needs to be addressed in order to provide comprehensive evidence-based nutrition guidance for Americans.

To address this research gap, we 1) estimated temporal trends (1999–2014) in DGA 2015–2020 recommended vegetable variety, 2) determinants of recommended vegetable variety, and 3) examined the relationship between recommended vegetable variety and prevalence of cardiometabolic disease. We additionally examined 4) temporal trends (1999–2014) in vegetable intake amount, 5) the relationship between vegetable amount and prevalence of cardiometabolic disease, and 6) the relationship between vegetable amount and recommended variety. All analyses were conducted overall and by sex.

## Methods

### Data on disease status and vegetable intake

Data on self-reported individual-level cardiometabolic disease status and other characteristics were acquired from the National Health and Nutrition Examination Survey (NHANES), waves 1999–2000, 2001–2002, 2003–2004, 2005–2006, 2007–2008, 2009–2010, 2011–2012, and 2013–2014 [15]. NHANES is a cross-sectional survey that collects data on health status and behaviors, as well as demography, from a sample of ~ 5000 individuals per year. Data are collected continuously and released in two-year cycles. Data on disease status were ascertained from responses to the question “Has a doctor or other health professional ever told you that you had [coronary heart disease/a stroke/diabetes]?” We evaluated each disease state separately as well as combined into cardiovascular disease (coronary heart disease or stroke) and cardiometabolic disease (cardiovascular disease or diabetes).

The dietary component of NHANES is What We Eat In America (WWEIA), for which individuals complete a 24-h recall administered by a trained interviewer using United States Department of Agriculture’s (USDA) Automated Multiple Pass Method [16]. The MyPyramid Equivalents Database (MPED) and the Food Patterns Equivalents Database (FPED) provide dietary data from WWEIA converted to cup-equivalents to standardize reported intake amounts across different vegetable types. Data on daily vegetable intake (including juice) were acquired from MPED 1.0 (applies to WWEIA 1999–2000 and 2001–2002) and 2.0 (applies to WWEIA 2003–2004); and FPED 2005–2006, 2007–2008, 2009–2010, 2011–2012, and 2013–2014. Intake data from day 1 only was used because this represents group-level intake [17].

### Measuring vegetable variety

The index used to measure vegetable variety was based on the Healthy Food Diversity index, which measures total dietary diversity independent of amount, and penalizes consumption of foods that are discordant with user-defined consumption targets [18]. The Healthy Food Diversity index is a general tool that can be applied to different populations by making only two minor modifications, which require the user to define the food groups to be included and to define the consumption targets for those food groups [19]. To measure vegetable variety, we modified the index to focus exclusively on consumption of vegetables, and we used consumption targets that reflect the DGA 2015–2020 vegetable subgroup recommendations (for 2200 kcal/day) for dark green vegetables, red and orange vegetables, legumes, starchy vegetables, and other vegetables [5]. The consumption targets of DGA 2015–2020 are in weekly units (i.e., cup-equivalents per week), so these were converted to daily units (recommended weekly consumption divided by seven) to be consistent with how intake data from WWEIA are measured.

The equation for the index is comprised of two parts. The first part is the Berry Index [20], which measures the number and proportionality of vegetable subgroups reported consumed by an individual. Values are bounded by 0 and 0.8, where the minimum score represents zero vegetable intake and the maximum score represents equal proportions of all vegetable subgroups. The Berry Index is expressed as:

$$ Berry\ Index=\left(1-\sum {o}_i^2\right), $$

where *o*_*i*_ is the observed proportion of each vegetable subgroup. The second part of the vegetable variety index is the Health Value [18], which assigns greater weighting to vegetable subgroups with greater recommended proportions. Values are bounded by 0.33 and 1, where the minimum value represents consumption of only the vegetable subgroup with the least weighting, and the maximum value represents consumption of only the vegetable subgroup with the greatest weighting. The Health Value is expressed as:

$$ Health\ Value=\left(\sum \left({r}_i\times {o}_i\right)/{o}_{max}\right), $$

where *r*_*i*_ is the recommended proportion of each vegetable subgroup and *o*_*max*_ is the maximum observed proportion out of all vegetable subgroups. Finally, the Healthy Food Diversity index is computed by multiplying the Berry Index by the Health Value, which ensures that higher index scores are achieved by: 1) consumption of more vegetable subgroups, and 2) greater relative consumption of vegetable subgroups that have greater weighting. The vegetable variety score, using the Healthy Food Diversity index, is bounded by 0 and 0.64.

### Statistical analyses

Trends in vegetable variety and vegetable amount from 1999 to 2014, overall and by sex, were evaluated with univariate linear regression models. To ensure that vegetable amount was uncorrelated with energy intake, vegetable amount was energy-adjusted to 2200 kcal/day using the residual method [21]. Sex differences in mean pooled vegetable variety scores and intake amounts were evaluated with two-sided Wald tests, and statistical significance was set at *P* < 0.05.

For analyses assessing the relationship between vegetable variety and cardiometabolic disease, individuals were grouped by quintile of vegetable variety scores, where individuals in quintile 1 had the lowest scores and individuals in quintile 5 had the greatest scores. For analyses investigating the relationship between vegetable amount and each cardiometabolic disease subtype, individuals were grouped by quintile of vegetable amount (energy-adjusted to 2200 kcal/day using the residual method [21]), where individuals in quintile 1 had the lowest intake amount and individuals in quintile 5 had the greatest intake amount. Differences in the odds of prevalent disease (cardiometabolic, cardiovascular, coronary heart, stroke, and diabetes) between quintiles of vegetable variety, and between quintiles of vegetable amount, were tested using multivariate logistic regression models. Linear trends across quintiles were tested using linear regression models. To examine the relationship between each of the vegetable amount subgroups (dark green vegetables, red and orange vegetables, legumes, starchy vegetables, and other vegetables) and prevalent disease, individuals were categorized dichotomously as either consumers (> 0 cup-equivalents/day) or non-consumers (0 cup-equivalents/day).

Diet-disease model covariates included age (20–30 y, 31–50 y, 51–70 y, > 70 y), sex, body mass index (BMI[kg/m^2^]; continuous), race/ethnicity (non-Hispanic white, non-Hispanic black, Mexican-American), education (<high school, high school or equivalent, some college, college graduate) income-to-poverty ratio (< 0.75, 0.75–1.24, 1.25–1.99, 2.0–3.99, ≥4.00), intake of fatty acids (unsaturated:saturated; continuous), and intake of added sugar (continuous). Intake of fatty acids and added sugar were energy-adjusted to 2200 kcal/day using the residual method [21]. Smoking status had a high degree of missingness (> 50%), so to include this important predictor variable in our models we categorized this variable as current smoker, non-current smoker, and missing. Models investigating the relationship between vegetable subgroups and prevalent disease were additionally adjusted for consumption amounts of all other subtypes of vegetables.

Multivariate ordered logistic regression models were used to assess determinants of vegetable variety; specifically, the relative odds of being in the next highest quintile of vegetable variety per one unit increase in the predictor variable. For dichotomous predictor variables, the referent group represents individuals who responded negatively to the survey question. For nominal predictor variables, the referent group represents individuals in the next less favorable category. Predictor variables were age (continuous), female (yes/no), BMI (continuous), education (<high school, high school or equivalent, some college, college graduate), income-to-poverty ratio (continuous), household size (number of persons; continuous), food security status (very low, low, marginal, full), food consumed away from home (meals per week; continuous), currently living with a domestic partner (yes/no), and current smoker (yes/no).

For all analyses, statistical significance was set at *P* < 0.05, and Bonferroni adjustment was used to correct for multiple pairwise comparisons. Stata15 (StataCorp; College Station, TX) was used for data management and analysis. All analyses were adjusted for the complex sampling design and sample weights of WWEIA data.

## Results

Individuals < 20 years of age (*n* = 36,859) and those not providing reliable dietary data or incomplete information on cardiometabolic disease status (*n* = 2678) were excluded from this analysis, resulting in a final analytic sample of 38,981 adults (Table 1). Most (39%) respondents were 31–50 years of age, and approximately one half (52%) were female. Most respondents were non-Hispanic white (78.5%) and attended some college (31%). The majority of respondents had an income-to-poverty ratio of ≥4.00 (36%) and a BMI of 25 to < 30 (53%). Most respondents did not report smoking status (53%), but a similar proportion reported currently smoking (23%) as not currently smoking (25%). Mean household size was 3 persons, and most (81%) respondents reported being fully food secure. Respondents reported consuming a mean of 3.4 meals per week away from home. Nearly two-thirds (63%) of respondents reported currently living with a domestic partner. Nearly 7000 respondents reported being told by a doctor or health professional that they had a cardiometabolic disease, including 1615 with coronary heart disease, 1430 who had a stroke, 5129 with diabetes, and 2805 with cardiovascular disease.

**Table 1:** Characteristics of study population, NHANES 1999–2014

Characteristic	n^a^	Percent (95% CI)^b^
Age (y)	38,981	
20–30		21.2 (20.2–22.2)
31–50		39.1 (38.0–40.1)
51–70		29.2 (28.3–30.0)
70+		10.6 (10.1–11.1)
Sex	38,981	
Women		52.0 (51.4–52.5)
Men		48.0 (47.5–48.6)
Race/ethnicity	33,528	
Non-Hispanic white		78.5 (76.4–80.4)
Non-Hispanic black		12.6 (11.2–14.1)
Mexican-American		8.9 (7.7–10.3)
Education	38,930	
Less than high school		18.3 (17.4–19.4)
High school or equivalent		24.1 (23.2–25.1)
Some college		30.9 (30.1–31.7)
College graduate		26.6 (25.2–28.1)
Income-to-poverty ratio	35,890	
< 0.75		9.1 (8.3–9.8)
0.75–1.24		11.7 (10.9–12.4)
1.25–1.99		14.7 (14.0–15.4)
2.00–3.99		28.9 (27.9–29.9)
4.00+		35.7 (34.1–37.4)
BMI (kg/m^2^)	38,712	
> 18.5		1.6 (1.4–1.8)
18.5–24.9		30.1 (29.2–31.0)
25 to < 30		52.8 (51.9–53.6)
≥ 30		15.5 (14.9–16.2)
Current smoker	38,981	
No		24.6 (23.7–25.4)
Yes		22.7 (21.9–23.6)
Missing		52.7 (51.6–53.8)
Household size (n)	38,981	3.0 (3.0–3.0)
Food security status	38,243	
Very low		5.0 (4.6–5.4)
Low		6.7 (6.3–7.2)
Marginal		7.6 (7.1–8.1)
Full		80.7 (79.8–81.5)
Food consumed away from home (meals/week)	38,964	3.4 (3.3–3.5)
Currently living with a domestic partner	38,499	
No		37.2 (36.1–38.4)
Yes		62.8 (61.6–63.9)
Prevalent disease^c^		
Coronary heart disease	1615	3.3 (3.1–3.6)
Stroke	1430	2.7 (2.5–2.9)
Diabetes	5129	9.8 (9.4–10.2)
Cardiovascular disease^d^	2805	5.6 (5.2–6.0)
Cardiometabolic disease^e^	6977	13.6 (13.0–14.2)

BMI, body mass index

^a^Sample sizes are unweighted

^b^Percentages within each column (adjusted for survey weight), unless otherwise specified

^c^Values represent the number of cases and the percent of cases out of the total sample

^d^Coronary heart disease or stroke

^e^Cardiovascular disease or diabetes

**Fig. 1:**
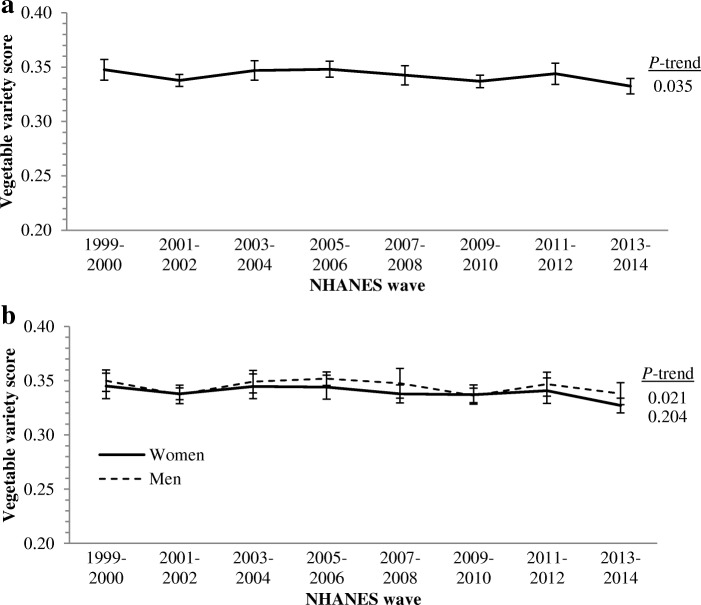
Trends in variety of vegetable intake among adults **a**) overall, and by **b**) sex, from 1999 to 2014. NHANES, National Health and Nutrition Examination Survey. Maximum possible variety score is 0.64. Mean (1999–2014) variety score for men = 34.5, women = 33.9, *P* for difference = 0.007. Sample sizes are: 38,981 overall, 20,270 women, 18,711 men

Overall, the mean daily vegetable variety score decreased from 0.35 (95% CI: 0.34–0.36) in 1999–2000 to 0.33 (0.33–0.34) in 2013–2014 (*P* = 0.035), out of a maximum possible score of 0.64 (Fig. 1a). Over this same time period, the variety of vegetables consumed decreased among men (*P* = 0.021) but not women (*P* = 0.204), yet men displayed higher vegetable variety scores (*P* = 0.007) when data were pooled across survey waves (Fig. 1b).

Respondents who reported currently living with a domestic partner had 22% greater odds (OR: 1.22, 95% CI: 1.15–1.30) of being in the next highest quintile of vegetable variety compared to those who reported not currently living with a domestic partner (Table 2). Respondents who reported currently smoking had 14% (0.86, 0.79–0.94) lower odds of being in the next highest quintile of vegetable variety compared to those who reported not currently smoking. Additional relationships between predictor variables and vegetable variety, but of lower magnitude, were observed for food security status (1.07, 1.03–1.11), income-to-poverty ratio (1.04, 1.02–1.07), food consumed away from home (1.03, 1.0.2–1.04), BMI (1.01, 1.00–1.01), and age (1.00, 1.00–1.00).

**Table 2:** Determinants of daily vegetable variety among adults, 1999–2014

Characteristic	Model 1 (*n* = 34,449)^a^		Model 2 (*n* = 16,119)^a,b^	
	Odds ratio (95% CI)	*P*	Odds ratio (95% CI)	*P*
Age (y)^c^	1.00 (1.00–1.00)	0.005	1.00 (0.99–1.00)	0.001
Female^d^	0.98 (0.93–1.03)	0.367	0.98 (0.91–1.05)	0.526
Education^e^	1.02 (0.99–1.05)	0.275	1.01 (0.97–1.05)	0.525
Income-to-poverty ratio^c^	1.04 (1.02–1.07)	0.001	1.04 (1.01–1.08)	0.015
BMI (kg/m^2^)^c^	1.01 (1.00–1.01)	0.010	1.00 (1.00–1.01)	0.388
Household size (n)^c^	0.99 (0.97–1.01)	0.183	0.98 (0.95–1.02)	0.308
Food security status^f^	1.07 (1.03–1.11)	< 0.001	1.05 (1.00–1.11)	0.049
Food consumed away from home (meals/week)^c^	1.03 (1.02–1.04)	< 0.001	1.03 (1.02–1.04)	< 0.001
Currently living with a domestic partner^d^	1.22 (1.15–1.30)	< 0.001	1.27 (1.16–1.39)	< 0.001
Current smoker^d^			0.86 (0.79–0.94)	0.001

^a^Ordered logistic regression predicting the relative odds of being in the next highest quintile of daily vegetable variety per one unit increase in the predictor variable. For dichotomous predictor variables, the referent group represents individuals who responded negatively to the survey question. For nominal predictor variables (education and food security status), the referent group represents individuals in the next less favorable category as defined in subsequent footnotes

^b^Model 1 + smoking status

^c^Continuous

^d^Yes/no.

^e^Less than high school, high school or equivalent, some college, college graduate

^f^Very low, low, marginal, full

**Fig. 2:**
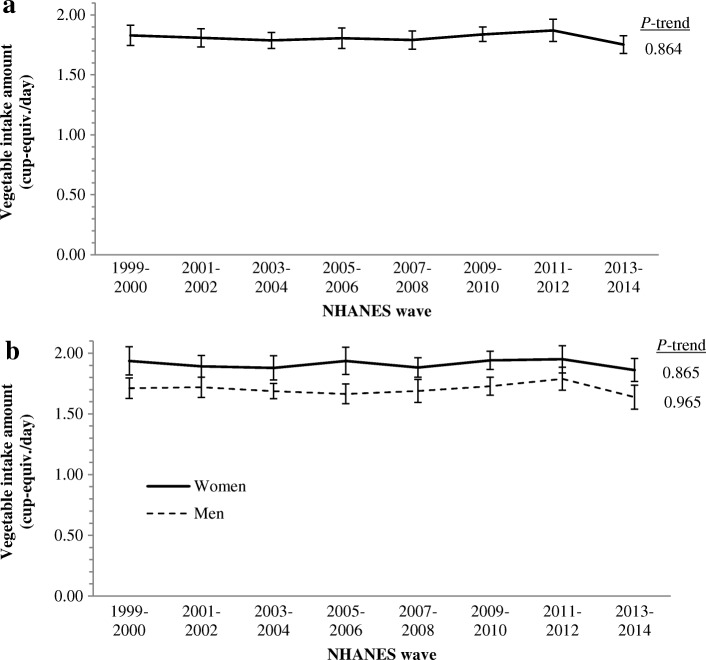
Trends in vegetable intake amount among adults **a**) overall, and by **b**) sex, from 1999 to 2014. NHANES, National Health and Nutrition Examination Survey. All data are energy-adjusted to 2200 kcal/day. Mean (1999–2014) energy-adjusted intake amount (cup-equiv./day) for men = 1.70 (1.67–1.73), women = 1.91 (1.87–1.94), *P* for difference < 0.001. Sample sizes are: 38,981 overall, 20,270 women, 18,711 men

No trend in daily vegetable amount was observed from 1999 to 2014 for both sexes combined (*P* = 0.864; Fig. 2a) or when men and women were examined separately (*P* = 0.965 for men, *P* = 0.865 for women; Fig. 2b). When the data were pooled across survey waves, women reported consuming greater (*P* < 0.001) amounts of vegetables (1.91 cup-equivalents, 95% CI: 1.87–1.94) than men (1.70, 1.67–1.73). By vegetable subgroups, overall (men and women combined) intake of starchy vegetables decreased (*P* < 0.001) and intake of dark green vegetables increased (*P* < 0.001) from 1999 to 2014, but no trends were observed for other subgroups; similar results were observed among men and women separately (Additional file 1: Figure S1).

Table 3 displays the overall odds of cardiometabolic disease prevalence, by disease subtype, between quintiles of vegetable variety. Compared to those in quintile 1 (least variety), no relationship was observed between greater vegetable variety and disease prevalence (*P* > 0.05 for all outcomes). However, when analyzed as a linear trend across quintiles, a significant relationship was observed for coronary heart disease (*P* = 0.032); a similar finding was observed for men (*P* = 0.046 for coronary heart disease; Additional file 2: Table S1) but not women (*P* > 0.05 for all outcomes; Additional file 3: Table S2).

**Table 3:** Relationship between vegetable intake variety and prevalence of cardiometabolic disease among adults, 1999–2014

Cardiometabolic disease outcome	Variety quintile 1	Variety quintile 2	Variety quintile 3	Variety quintile 4	Variety quintile 5	*P*-trend
Odds ratio (95% CI)						
Cardiometabolic	Referent	0.85 (0.72–1.01)	0.98 (0.82–1.16)	0.97 (0.82–1.15)	1.00 (0.84–1.19)	0.496
Cardiovascular	Referent	0.90 (0.69–1.18)	0.93 (0.71–1.20)	0.81 (0.63–1.05)	0.85 (0.64–1.12)	0.145
Coronary heart	Referent	0.93 (0.67–1.28)	0.91 (0.65–1.27)	0.73 (0.54–1.01)	0.76 (0.54–1.08)	0.032
Stroke	Referent	0.91 (0.66–1.25)	0.97 (0.70–1.34)	0.94 (0.69–1.29)	1.00 (0.75–1.35)	0.882
Diabetes	Referent	0.82 (0.68–0.98)	0.93 (0.77–1.13)	0.99 (0.82–1.19)	1.04 (0.86–1.26)	0.198

Adjusted for age, sex, body mass index, smoking status, race/ethnicity, intake of fatty acids (unsaturated:saturated), intake of added sugar, income-to-poverty ratio, and education

Maximum possible variety score is 64

Median vegetable variety scores for each quintile are: quintile 1 = 0, quintile 2 = 0.17, quintile 3 = 0.33, quintile 4 = 0.43, quintile 5 = 0.52

Cardiometabolic disease includes coronary heart disease, stroke, and diabetes

Cardiovascular disease includes coronary heart disease and stroke

Table 4 displays the overall odds of cardiometabolic disease prevalence, by disease subtype, between quintiles of vegetable amount. Compared to individuals in quintile 1 (least amount), no relationship was observed between greater vegetable intake and disease prevalence (*P* > 0.05 for all outcomes). When analyzed as a linear trend across quintiles, greater vegetable intake was associated with lower odds of having coronary heart disease (*P* = 0.026), but no relationships were observed between vegetable amount and other cardiometabolic disease subtypes. No relationship between vegetable amount and disease subtypes was observed among men (Additional file 4: Table S3). Among women, individuals with the greatest vegetable intake (quintile 5) had lower odds (0.65, 95% CI: 0.49–0.88) of having cardiovascular disease, but no linear relationships were observed between vegetable amount and disease subtypes (Additional file 5: Table S4).

**Table 4:** Relationship between vegetable intake amount and prevalence of cardiometabolic disease among adults, 1999–2014

Cardiometabolic disease outcome	Amount quintile 1	Amount quintile 2	Amount quintile 3	Amount quintile 4	Amount quintile 5	*P*-trend
Odds ratio (95% CI)						
Cardiometabolic	Referent	0.88 (0.76–1.03)	1.04 (0.88–1.21)	0.94 (0.82–1.06)	0.95 (0.82–1.11)	0.805
Cardiovascular	Referent	0.79 (0.65–0.97)	0.97 (0.78–1.21)	0.81 (0.67–0.99)	0.79 (0.64–0.97)	0.050
Coronary heart	Referent	0.84 (0.65–1.07)	0.90 (0.70–1.16)	0.80 (0.64–1.01)	0.75 (0.59–0.96)	0.026
Stroke	Referent	0.75 (0.55–1.02)	1.08 (0.82–1.43)	0.88 (0.70–1.11)	0.87 (0.67–1.12)	0.667
Diabetes	Referent	0.97 (0.82–1.14)	1.04 (0.87–1.25)	0.98 (0.85–1.13)	1.00 (0.86–1.17)	0.961

Adjusted for age, sex, body mass index, smoking status, race/ethnicity, intake of fatty acids (unsaturated:saturated), intake of added sugar, income-to-poverty ratio, and education

Median energy-adjusted vegetable intake (cup-equivalents/day) for each quintile is: quintile 1 = 0, quintile 2 = 0.95, quintile 3 = 1.61, quintile 4 = 1.89, quintile 5 = 2.10

Cardiometabolic disease includes coronary heart disease, stroke, and diabetes

Cardiovascular disease includes coronary heart disease and stroke

Overall, individuals who reported consuming dark green vegetables on the day of the survey (i.e., consumers) had lower odds of having cardiovascular disease (0.86, 95% CI: 0.74–0.99) and coronary heart disease (0.78, 0.65–0.94) compared to individuals who reported not consuming any green vegetables (i.e., non-consumers; Table 5); but no association was observed when stratified by sex (Additional file 6: Table S5 and Additional file 7: Table S6). Among men, consumers of starchy vegetables had greater odds of having diabetes (1.20, 1.04–1.38) compared to non-consumers (Additional file 6: Table S5). Among women, no associations were observed between consumption of vegetable subtypes and prevalent disease (Additional file 7: Table S6).

**Table 5:** Relationship between vegetable intake amount and prevalence of cardiometabolic disease among adults, 1999–2014

Cardiometabolic disease outcome	Vegetable subtypes				
	Dark green	Red/orange	Legumes	Starchy	Other
Odds ratio (95% CI)					
Cardiometabolic					
Non-consumers	Referent	Referent	Referent	Referent	Referent
Consumers	0.91 (0.87–1.09)	0.97 (0.87–1.09)	0.97 (0.85–1.10)	1.07 (0.98–1.17)	1.01 (0.91–1.12)
Cardiovascular					
Non-consumers	Referent	Referent	Referent	Referent	Referent
Consumers	0.86 (0.74–0.99)*	0.90 (0.77–1.04)	1.02 (0.87–1.2)	1.02 (0.89–1.16)	0.94 (0.81–1.10)
Coronary heart					
Non-consumers	Referent	Referent	Referent	Referent	Referent
Consumers	0.78 (0.65–0.94)*	0.87 (0.72–1.05)	1.18 (0.97–1.44)	0.96 (0.81–1.12)	0.96 (0.78–1.19)
Stroke					
Non-consumers	Referent	Referent	Referent	Referent	Referent
Consumers	0.96 (0.76–1.21)	0.96 (0.76–1.21)	0.82 (0.66–1.03)	1.07 (0.92–1.23)	0.94 (0.78–1.14)
Diabetes					
Non-consumers	Referent	Referent	Referent	Referent	Referent
Consumers	0.92 (0.81–1.05)	1.05 (0.93–1.18)	0.96 (0.83–1.11)	1.10 (1.00–1.21)	1.03 (0.93–1.15)

Adjusted for age, sex, body mass index, smoking status, race/ethnicity, intake of fatty acids (unsaturated:saturated), intake of added sugar, income-to-poverty ratio, education, and the consumption amount of the remaining vegetable subtypes

*P < 0.05

Cardiometabolic disease includes coronary heart disease, stroke, and diabetes

Cardiovascular disease includes coronary heart disease and stroke

The relationship between vegetable amount and variety is displayed in Table 6. Vegetable variety was positively associated with vegetable amount overall (*P* < 0.001) and by sex (*P* < 0.001 for both sexes).

**Table 6:** Mean vegetable intake amount by quintile of vegetable variety score, overall and by sex, 1999–2014

Vegetable intake variety	Mean cup-equivalents (95% CI)	*P*-trend
Overall		< 0.001
Quintile 1	0.62 (0.57–0.68)	
Quintile 2	1.23 (1.19–1.28)	
Quintile 3	1.80 (1.76–1.84)	
Quintile 4	2.11 (2.06–2.15)	
Quintile 5	2.36 (2.31–2.41)	
Men		< 0.001
Quintile 1	0.54 (0.48–0.60)	
Quintile 2	1.19 (1.13–1.25)	
Quintile 3	1.69 (1.63–1.75)	
Quintile 4	1.93 (1.88–1.97)	
Quintile 5	2.23 (2.17–2.29)	
Women		< 0.001
Quintile 1	0.69 (0.61–0.78)	
Quintile 2	1.28 (1.21–1.34)	
Quintile 3	1.90 (1.84–1.96)	
Quintile 4	2.27 (2.21–2.34)	
Quintile 5	2.48 (2.41–2.56)	

Adjusted for age, sex, body mass index, smoking status, race/ethnicity, intake of fatty acids (unsaturated:saturated), intake of added sugar, income-to-poverty ratio, and education

Median vegetable variety scores for each quintile are: quintile 1 = 0, quintile 2 = 0.17, quintile 3 = 0.33, quintile 4 = 0.43, quintile 5 = 0.52

## Discussion

This is the first study, to the best of our knowledge, to measure the association between DGA-recommended vegetable variety and amount and prevalent cardiometabolic disease. In this study of nearly 40 thousand US adults over a 16-year period, we observed low vegetable variety, and an overall negative temporal trend was statistically significant but not clinically meaningful. Domestic partner status and smoking status were strongly associated with vegetable variety. No overall temporal trend was observed for vegetable amount. We also observed an inverse relationship between variety of vegetable intake and prevalent coronary heart disease overall and among men, but not women; and we found an inverse relationship between vegetable amount and prevalent coronary heart disease overall but not for either sex independently. No diet-disease relationships were observed for prevalent cardiometabolic disease, cardiovascular disease, stroke, or diabetes.

This work extends the line of research linking dietary exposures to cardiometabolic outcomes by demonstrating that vegetable variety and amount are independently associated with prevalent coronary heart disease among the US adult general population. In contrast to previous studies which observed no relationship between variety of vegetable intake and coronary heart disease [12, 13], we observed a relationship overall and for men, but not women. This discrepancy may be due to differences in the measurement of vegetable variety between studies: others measured variety as the number of distinct fruits and vegetables consumed [12, 13], whereas we measured it as adherence to the DGA 2015–2020 recommendations which are based on vegetable subgroups. It also cannot be ruled out that this discrepancy may be due to mis-categorization of vegetable variety scores in the present study, resulting from a lack of repeated measures of dietary intake. Yet to the best of our knowledge, the present study is the first to examine the relationship between adherence to the DGA 2015–2020 vegetable variety intake recommendations and prevalent cardiometabolic disease among the US adult general population.

In the present study, domestic partner status had a stronger relationship with vegetable variety than other socio-demographic and lifestyle behaviors we examined. Findings from the EPIC cohort demonstrated that being single, widowed, or not having a domestic partner was associated with lower vegetable variety [22], and that marital transitions (becoming separated, divorced, or widowed) were associated with reduced vegetable variety [23]; and similar findings were observed in US populations [24, 25]. Social contact, and particularly close personal relationships, can affect vegetable intake in several ways. On a pragmatic level, living alone can reduce economies of scale in household food preparation, especially for raw vegetables that may require greater amounts of processing (washing, slicing, cooking) than other foods [22]. On a psycho-social level, socialization provides individuals with a sense of motivating self-worth that encourages health-promoting behaviors, and also provides the social support to adopt shared norms and responsibility for overall health and well-being [23].

Current smokers were less likely to consume a greater variety of vegetables compared to non-smokers in the present study. Others have reported that fruit and vegetable intake is inversely associated with the number of daily cigarettes smoked, time to first cigarette, and nicotine dependence [26, 27]. Reduced vegetable variety among smokers is likely attributable to reduced taste sensitivity from chronic exposure to cigarette smoke [28, 29], and may also be attributable to a generally unhealthy lifestyle pattern that includes multiple risk factors for chronic disease [27, 30].

We observed that respondents who reported consuming dark green vegetables on the day of the dietary survey had lower odds of having cardiovascular disease and coronary heart disease compared to non-consumers, which is in contrast to others [12]. This difference may be attributable to greater heterogeneity in the dietary exposure of other studies which included green fruits (such as kiwi and honeydew melon) in the same category as green vegetables, and may also be due to differences in residual confounding associated with the disparate study populations (American vs. Dutch). We also observed that intake of dark green vegetables has increased from 1999 to 2014, which is in accord with others [31]. Taken together, the increasing trend of dark-green vegetable intake and the relationship between dark-green vegetable intake and prevalent cardiovascular disease and coronary heart disease indicates one way that Americans are improving their diet in cardioprotective ways.

Vegetables are low energy-dense foods and contain a number of bioactive compounds that are linked with reduced risk of cardiometabolic disease incidence and mortality, including dietary fiber, vitamins, minerals, and phytochemicals [1, 32]. Vegetable consumption reduces cardiometabolic risk through multiple mechanisms attributed to these bioactive compounds, including improvement of lipoprotein profiles, reduced blood pressure, inhibited platelet aggregation, increased insulin sensitivity, and reduced inflammation and oxidant stress [33–35]. Carotenoids, pigments found in dark-green and red and orange vegetables, are inversely associated with markers of detrimental inflammation and oxidative stress [36, 37], and tomato juice, rich in lycopene, has been reported to decrease LDL cholesterol amount and oxidation [38]. Carotenoids may also be associated with decreased and modified adiposity [39, 40]. Dark-green vegetables are also rich sources of nitrate, comprising ~ 80% of daily exposure; nitrate is important for the production of nitric oxide and may be cardioprotective [41]. Spinach, red pepper, beets, and broccoli are also rich in phenolic compounds and have antioxidant activity, and many polyphenols are anti-atherogenic [42, 43]. Flavonoids, a polyphenol also found in vegetables [44], are associated with decreased inflammation [45]. Folate is found in dark-green vegetables and beans and peas and is cardioprotective. Most vegetables are good sources of potassium, a nutrient of public health concern, which is important in blood pressure homeostasis [46].

In addition to ensuring adequate intake of health-promoting bioactive compounds, consuming a variety of vegetables may impact health status through mediated mechanisms. In accordance with other studies [10–12], we observed a strong relationship between vegetable variety and amount, which is important because vegetable amount has been strongly linked to reduce risk of cardiometabolic disease mortality [2–4]. Increased vegetable variety can increase liking [9], perhaps through reduced habituation, which in turn can increase the overall amount consumed [10–12]. Therefore, the recommendation to consume a variety of vegetables is still a potent message with important health implications for the general public. Clinicians should continue to encourage increased vegetable variety and amount to protect against cardiometabolic disease.

The strengths of this study include its large sample size and national representativeness, which makes our findings generalizable to the US adult population. Because of known sex differences in disease prevalence and food intake patterns [47, 48], we also stratified our results to better understand sex differences in our outcomes of interest. Importantly, we tied the concept of variety to the DGA vegetable subgroups, which may be more relevant to health than other types of variety measures [49].

Several limitations also warrant mention. Foremost, this study did not include repeated measures of dietary exposure. Although a single dietary recall is representative of usual intake at the group level, it is not a reliable estimate of usual intake at the individual level [17], so mis-categorization of vegetable consumption patterns cannot be ruled out. Relatedly, although the DGA 2015–2020 vegetable variety recommendations are provided in weekly units (i.e., cup-equivalents per week), we converted these to daily units to be consistent with how WWEIA data are reported. This may have resulted in an underestimate of vegetable variety scores because individuals likely consume greater variety over the course of a week than a single day, which could also have led to mis-categorization. However, we do not expect that this led to bias in our estimates because there is no indication that this differentially affected the variety scores between individuals with different cardiometabolic disease status. Self-reported dietary data are subject to measurement error, and individuals may over-report consumption of perceived healthy foods like vegetables; yet self-reported data are nonetheless useful for comparing dietary patterns between groups [50]. The cross-sectional design of this study precludes any determination of a causal relationship between vegetable intake patterns and prevalent cardiometabolic disease. Indeed, it is plausible that individuals who were told by their physician that they had cardiometabolic disease subsequently increased their vegetable intake. Yet if disease diagnosis stimulated healthful dietary change, we might expect to observe that individuals with prevalent disease had greater intake of vegetables compared to their disease-free counterparts; but this was not observed in the present study. Nonetheless, we cannot make causal inference from these data, and longitudinal studies are needed to determine a causal relationship between vegetable intake patterns and disease incidence. Additional large-scale longitudinal studies with repeated measures of dietary exposure, with disease cases adjudicated by trained health professionals, are needed to address the relationship between vegetable variety and risk of cardiometabolic disease incidence and mortality.

## Conclusion

This is the first study, to the best of our knowledge, to measure the association between DGA-recommended vegetable variety and amount and prevalent cardiometabolic disease subtypes. Vegetable variety and amount were inversely associated with prevalent coronary heart disease among our sample of nearly 40 thousand US adults over a 16-year period. Vegetable variety was strongly associated with vegetable amount, likely mediated by reduced habituation and increased liking. Vegetable variety was also strongly associated with domestic partner status and smoking status, which adds further support for the health-promoting effects of social contact, and highlights the additional unhealthy lifestyle behaviors associated with smoking. Vegetables are rich in key bioactive components that are associated with positive health outcomes. Increasing vegetable variety is still an important message for the public, and clinicians should continue to encourage both increased vegetable variety and amount.

## Additional files

Additional file 1: **Figure S1.** Trends in vegetable intake amount among adults a) overall, b) among females, and c) among males, from 1999 to 2014. (PDF 162 kb)

Additional file 2: **Table S1.** Relationship between DGA vegetable intake variety and prevalence of cardiometabolic disease among men, 1999–2014. (PDF 55 kb)

Additional file 3: **Table S2.** Relationship between DGA vegetable intake variety and prevalence of cardiometabolic disease among women, 1999–2014. (PDF 54 kb)

Additional file 4: **Table S3.** Relationship between vegetable intake amount and prevalence of cardiometabolic disease among men, 1999–2014. (PDF 49 kb)

Additional file 5: **Table S4.** Relationship between vegetable intake amount and prevalence of cardiometabolic disease among women, 1999–2014. (PDF 51 kb)

Additional file 6: **Table S5.** Relationship between vegetable intake amount and prevalence of cardiometabolic disease among men, 1999–2014. (PDF 61 kb)

Additional file 7: **Table S6.** Relationship between vegetable intake amount and prevalence of cardiometabolic disease among women, 1999–2014. (PDF 60 kb)

## Abbreviations

24HR: 24-h recall
BMI: Body Mass Index
DGA: Dietary Guidelines for Americans
DRI: Dietary Reference Intakes
FPED: Food Patterns Equivalents Database
MPED: My Pyramid Equivalents Database
NHANES: National Health and Nutrition Examination Survey
USDA: United States Department of Agriculture
WWEIA: What We Eat In America
