# Supplementary material for: Greater vegetable variety and amount are associated with lower prevalence of coronary heart disease: National Health and Nutrition Examination Survey, 1999–2014
Source: Nutr J. 2018 Jul 10;17:67. doi: 10.1186/s12937-018-0376-4 (PMC6040059; doi:10.1186/s12937-018-0376-4)
Supplement: Supplementary file 1 — Figure S1. Trends in vegetable intake amount among adults a) overall, b) among females, and c) among males, from 1999 to 2014. (PDF 162 kb) [file 12937_2018_376_MOESM1_ESM.pdf]

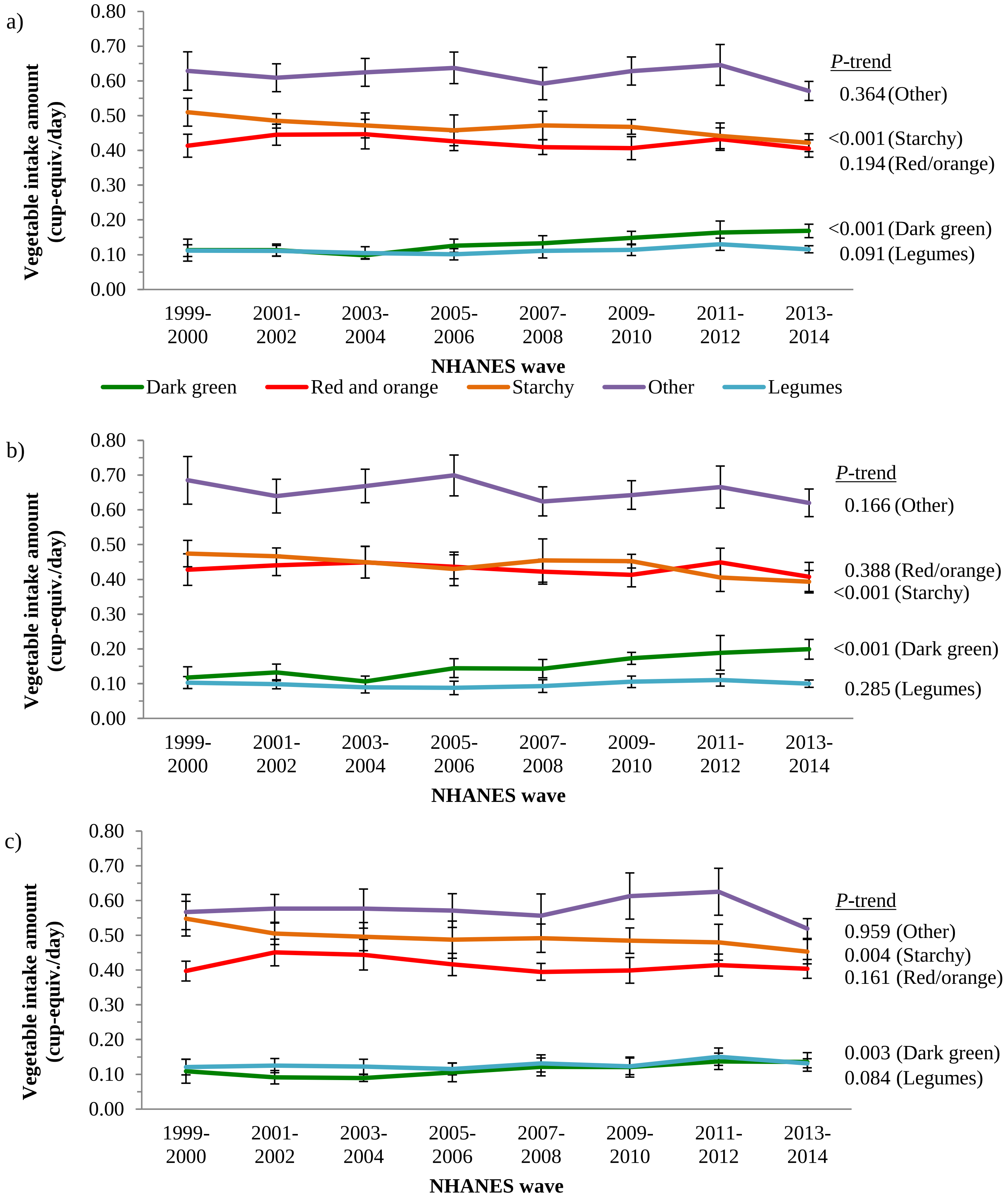

Figure S1: Trends in vegetable intake amount among adults a) overall, b) among females, and c) among males, from 1999-2014

NHANES, National Health and Nutrition Examination Survey
All data are energy-adjusted to 2,200 kcal/day.
