# Supplementary material for: Greater vegetable variety and amount are associated with lower prevalence of coronary heart disease: National Health and Nutrition Examination Survey, 1999–2014
Source: Nutr J. 2018 Jul 10;17:67. doi: 10.1186/s12937-018-0376-4 (PMC6040059; doi:10.1186/s12937-018-0376-4)
Supplement: Supplementary file 2 — Table S1. Relationship between DGA vegetable intake variety and prevalence of cardiometabolic disease among men, 1999–2014. (PDF 55 kb) [file 12937_2018_376_MOESM2_ESM.pdf]

Table S1: Relationship between DGA vegetable intake variety and prevalence of cardiometabolic disease among men, 1999-2014

Cardiometabolic disease outcome	Variety quintile 1	Variety quintile 2	Variety quintile 3	Variety quintile 4	Variety quintile 5	*P* -trend
			Odds ratio (95% CI)			
Cardiometabolic	Referent	0.87 (0.68-1.11)	0.97 (0.76-1.25)	1.07 (0.85-1.36)	1.03 (0.81-1.31)	0.311
Cardiovascular	Referent	0.87 (0.60-1.24)	0.92 (0.66-1.28)	0.81 (0.56-1.17)	0.81 (0.55-1.18)	0.237
Coronary heart	Referent	0.75 (0.50-1.11)	0.79 (0.54-1.18)	0.63 (0.41-0.96)	0.66 (0.42-1.04)	0.046
Stroke	Referent	1.16 (0.70-1.94)	1.17 (0.73-1.88)	1.27 (0.78-2.07)	1.23 (0.76-1.98)	0.328
Diabetes	Referent	0.81 (0.82-1.30)	0.89 (0.68-1.17)	1.13 (0.87-1.46)	1.06 (0.80-1.42)	0.134

Adjusted for age, body mass index, smoking status, race/ethnicity, intake of fatty acids (unsaturated:saturated), intake of added sugar, income-to-poverty ratio, and education.

Maximum possible variety score is 64.

Median vegetable diversity scores for each quintile are: quintile 1=0, quintile2=0.17, quintile 3=0.33, quintile 4=0.43, quintile 5=0.52

Cardiometabolic disease includes coronary heart disease, stroke, and diabetes.

Cardiovascualr disease includes coronary heart disease and stroke.
