# Supplementary material for: Greater vegetable variety and amount are associated with lower prevalence of coronary heart disease: National Health and Nutrition Examination Survey, 1999–2014
Source: Nutr J. 2018 Jul 10;17:67. doi: 10.1186/s12937-018-0376-4 (PMC6040059; doi:10.1186/s12937-018-0376-4)
Supplement: Supplementary file 3 — Table S2. Relationship between DGA vegetable intake variety and prevalence of cardiometabolic disease among women, 1999–2014. (PDF 54 kb) [file 12937_2018_376_MOESM3_ESM.pdf]

Table S2: Relationship between DGA vegetable intake variety and prevalence of cardiometabolic disease among women, 1999-2014

Cardiometabolic disease outcome	Variety quintile 1	Variety quintile 2	Variety quintile 3	Variety quintile 4	Variety quintile 5	*P* -trend
	Odds ratio (95% CI)					
Cardiometabolic	Referent	0.83 (0.66-1.05)	0.98 (0.77-1.25)	0.89 (0.68-1.16)	0.97 (0.75-1.26)	0.974
Cardiovascular	Referent	0.94 (0.68-1.29)	0.94 (0.68-1.30)	0.84 (0.60-1.17)	0.89 (0.65-1.23)	0.349
Coronary heart	Referent	1.38 (0.88-2.16)	1.21 (0.77-1.90)	1.04 (0.65-1.66)	1.00 (0.61-1.63)	0.589
Stroke	Referent	0.78 (0.54-1.11)	0.86 (0.58-1.29)	0.79 (0.52-1.19)	0.89 (0.61-1.29)	0.593
Diabetes	Referent	0.82 (0.63-1.06)	0.96 (0.72-1.28)	0.87 (0.65-1.17)	1.02 (0.76-1.35)	0.766

Adjusted for age, body mass index, smoking status, race/ethnicity, intake of fatty acids (unsaturated:saturated), intake of added sugar, income-to-poverty ratio, and education.

Maximum possible variety score is 64.

Median vegetable diversity scores for each quintile are: quintile 1=0, quintile2=0.17, quintile 3=0.33, quintile 4=0.43, quintile 5=0.52

Cardiometabolic disease includes coronary heart disease, stroke, and diabetes.

Cardiovascualr disease includes coronary heart disease and stroke.
