# Supplementary material for: Greater vegetable variety and amount are associated with lower prevalence of coronary heart disease: National Health and Nutrition Examination Survey, 1999–2014
Source: Nutr J. 2018 Jul 10;17:67. doi: 10.1186/s12937-018-0376-4 (PMC6040059; doi:10.1186/s12937-018-0376-4)
Supplement: Supplementary file 4 — Table S3. Relationship between vegetable intake amount and prevalence of cardiometabolic disease among men, 1999–2014. (PDF 49 kb) [file 12937_2018_376_MOESM4_ESM.pdf]

Table S3: Relationship between vegetable intake amount and prevalence of cardiometabolic disease among men, 1999-2014

Cardiometabolic disease outcome	Amount quintile 1	Amount quintile 2	Amount quintile 3	Amount quintile 4	Amount quintile 5	*P* -trend
	-------------------------------		Odds ratio (95% CI)	-------------------------------		
Cardiometabolic	Referent	0.91 (0.73-1.13)	1.00 (0.80-1.24)	1.00 (0.83-1.22)	1.04 (0.83-1.31)	0.489
Cardiovascular	Referent	0.76 (0.58-1.00)	0.88 (0.67-1.16)	0.78 (0.60-1.00)	0.81 (0.61-1.07)	0.188
Coronary heart	Referent	0.81 (0.59-1.10)	0.90 (0.66-1.25)	0.77 (0.57-1.04)	0.79 (0.58-1.08)	0.149
Stroke	Referent	0.72 (0.49-1.06)	0.91 (0.62-1.34)	0.78 (0.54-1.14)	0.86 (0.58-1.27)	0.615
Diabetes	Referent	1.01 (0.78-1.29)	1.03 (0.80-1.32)	1.08 (0.88-1.33)	1.12 (0.88-1.42)	0.314

Adjusted for age, body mass index, smoking status, race/ethnicity, intake of fatty acids (unsaturated:saturated), intake of added sugar, income-to-poverty ratio, and education.

Median energy-adjusted vegetable intake (cup-equivalents/day) for each quintile is: quintile 1=0, quintile2=0.95, quintile 3=1.67, quintile 4=2.00, quintile 5=2.18

Cardiometabolic disease includes coronary heart disease, stroke, and diabetes.

Cardiovascualr disease includes coronary heart disease and stroke.
