# Supplementary material for: Greater vegetable variety and amount are associated with lower prevalence of coronary heart disease: National Health and Nutrition Examination Survey, 1999–2014
Source: Nutr J. 2018 Jul 10;17:67. doi: 10.1186/s12937-018-0376-4 (PMC6040059; doi:10.1186/s12937-018-0376-4)
Supplement: Supplementary file 5 — Table S4. Relationship between vegetable intake amount and prevalence of cardiometabolic disease among women, 1999–2014. (PDF 51 kb) [file 12937_2018_376_MOESM5_ESM.pdf]

Table S4: Relationship between vegetable intake amount and prevalence of cardiometabolic disease among women, 1999-2014

Cardiometabolic disease outcome	Amount quintile 1	Amount quintile 2	Amount quintile 3	Amount quintile 4	Amount quintile 5	*P* -trend
	---------------------------------		Odds ratio (95% CI)	---------------------------------		
Cardiometabolic	Referent	0.86 (0.71-1.04)	1.09 (0.88-1.33)	0.88 (0.72-1.06)	0.89 (0.73-1.08)	0.360
Cardiovascular	Referent	0.86 (0.67-1.11)	0.93 (0.72-1.21)	0.74 (0.56-0.98)	0.65 (0.49-0.88)*	0.180
Coronary heart	Referent	0.89 (0.62-1.28)	0.90 (0.62-1.31)	0.85 (0.58-1.25)	0.71 (0.51-1.00)	0.078
Stroke	Referent	0.78 (0.52-1.17)	1.26 (0.87-1.83)	0.97 (0.71-1.33)	0.89 (0.63-1.24)	0.961
Diabetes	Referent	0.93 (0.75-1.17)	1.06 (0.85-1.34)	0.88 (0.72-1.09)	0.91 (0.74-1.12)	0.362

Adjusted for age, body mass index, smoking status, race/ethnicity, intake of fatty acids (unsaturated:saturated), intake of added sugar, income-to-poverty ratio, and education.

Median energy-adjusted vegetable intake (cup-equivalents/day) for each quintile is: quintile 1=0, quintile2=0.95, quintile 3=1.56, quintile 4=1.78, quintile 5=2.01

Cardiometabolic disease includes coronary heart disease, stroke, and diabetes.

Cardiovascualr disease includes coronary heart disease and stroke.

**P* <0.05 (Bonferroni adjusted)
