# Supplementary material for: Greater vegetable variety and amount are associated with lower prevalence of coronary heart disease: National Health and Nutrition Examination Survey, 1999–2014
Source: Nutr J. 2018 Jul 10;17:67. doi: 10.1186/s12937-018-0376-4 (PMC6040059; doi:10.1186/s12937-018-0376-4)
Supplement: Supplementary file 6 — Table S5. Relationship between vegetable intake amount and prevalence of cardiometabolic disease among men, 1999–2014. (PDF 61 kb) [file 12937_2018_376_MOESM6_ESM.pdf]

Table S5: Relationship between vegetable intake amount and prevalence of cardiometabolic disease among men, 1999-2014

Cardiometabolic disease outcome	Vegetable subtypes				
	Dark green	Red/orange	Legumes	Starchy	Other
	Odds ratio (95% CI)				
Cardiometabolic					
Non-consumers	Referent	Referent	Referent	Referent	Referent
Consumers	0.95 (0.83-1.13)	0.97 (0.83-1.13)	0.99 (0.83-1.18)	1.11 (0.97-1.26)	0.96 (0.81-1.13)
Cardiovascular					
Non-consumers	Referent	Referent	Referent	Referent	Referent
Consumers	0.87 (0.7-1.07)	0.83 (0.68-1.01)	1.08 (0.86-1.36)	0.98 (0.81-1.19)	0.94 (0.75-1.18)
Coronary heart					
Non-consumers	Referent	Referent	Referent	Referent	Referent
Consumers	0.78 (0.60-1.01)	0.81 (0.64-1.02)	1.19 (0.90-1.56)	0.89 (0.71-1.10)	0.98 (0.74-1.30)
Stroke					
Non-consumers	Referent	Referent	Referent	Referent	Referent
Consumers	1.06 (0.76-1.49)	1.06 (0.76-1.49)	0.88 (0.63-1.22)	1.12 (0.88-1.42)	0.88 (0.62-1.24)
Diabetes					
Non-consumers	Referent	Referent	Referent	Referent	Referent
Consumers	0.98 (0.8-1.19)	1.03 (0.86-1.24)	1.00 (0.83-1.20)	1.20 (1.04-1.38)*	0.95 (0.81-1.12)

Adjusted for age, sex, body mass index, smoking status, race/ethnicity, intake of fatty acids (unsaturated:saturated), intake of added sugar, income-to-poverty ratio, education, and the consumption amount of the remaining vegetable subtypes.

Cardiometabolic disease includes coronary heart disease, stroke, and diabetes.

Cardiovascualr disease includes coronary heart disease and stroke.

*P <0.05
