# Supplementary material for: Greater vegetable variety and amount are associated with lower prevalence of coronary heart disease: National Health and Nutrition Examination Survey, 1999–2014
Source: Nutr J. 2018 Jul 10;17:67. doi: 10.1186/s12937-018-0376-4 (PMC6040059; doi:10.1186/s12937-018-0376-4)
Supplement: Supplementary file 7 — Table S6. Relationship between vegetable intake amount and prevalence of cardiometabolic disease among women, 1999–2014. (PDF 60 kb) [file 12937_2018_376_MOESM7_ESM.pdf]

Table S6: Relationship between vegetable intake amount and prevalence of cardiometabolic disease among women, 1999-2014

Cardiometabolic disease outcome	Vegetable subtypes				
	Dark green	Red/orange	Legumes	Starchy	Other
	Odds ratio (95% CI)				
Cardiometabolic					
Non-consumers	Referent	Referent	Referent	Referent	Referent
Consumers	0.90 (0.84-1.17)	0.99 (0.84-1.17)	0.95 (0.80-1.13)	1.03 (0.91-1.17)	1.07 (0.92-1.24)
Cardiovascular					
Non-consumers	Referent	Referent	Referent	Referent	Referent
Consumers	0.86 (0.7-1.06)	0.98 (0.79-1.21)	0.97 (0.79-1.18)	1.05 (0.91-1.21)	0.94 (0.75-1.18)
Coronary heart					
Non-consumers	Referent	Referent	Referent	Referent	Referent
Consumers	0.80 (0.58-1.12)	0.99 (0.73-1.34)	1.21 (0.89-1.64)	1.09 (0.88-1.35)	0.92 (0.67-1.28)
Stroke					
Non-consumers	Referent	Referent	Referent	Referent	Referent
Consumers	0.91 (0.69-1.20)	0.91 (0.69-1.2)	0.79 (0.59-1.05)	1.02 (0.83-1.27)	1.01 (0.77-1.32)
Diabetes					
Non-consumers	Referent	Referent	Referent	Referent	Referent
Consumers	0.89 (0.75-1.06)	1.07 (0.89-1.29)	0.93 (0.77-1.13)	1.01 (0.88-1.16)	1.11 (0.94-1.30)

Adjusted for age, sex, body mass index, smoking status, race/ethnicity, intake of fatty acids (unsaturated:saturated), intake of added sugar, income-to-poverty ratio, education, and the consumption amount of the remaining vegetable subtypes.

Cardiometabolic disease includes coronary heart disease, stroke, and diabetes.

Cardiovascualr disease includes coronary heart disease and stroke.

*P <0.05
